# Beneficial Effects of Small Molecule Oligopeptides Isolated from *Panax ginseng* Meyer on Pancreatic Beta-Cell Dysfunction and Death in Diabetic Rats

**DOI:** 10.3390/nu9101061

**Published:** 2017-09-26

**Authors:** Meihong Xu, Bin Sun, Di Li, Ruixue Mao, Hui Li, Yong Li, Junbo Wang

**Affiliations:** 1 Department of Nutrition and Food Hygiene, School of Public Health, Peking University, Beijing 100191, China; xumeihong@bjmu.edu.cn (M.X.); berseraphim@bjmu.edu.cn (B.S.); lidiyy@163.com (D.L.); rx334@163.com (R.M.); huixin6101@163.com (H.L.); liyongbmu@163.com (Y.L.); 2 Beijing Key Laboratory of Toxicological Research and Risk Assessment for Food Safety, Peking University, Beijing 100191, China

**Keywords:** *Panax ginseng* oligopeptide, pancreatic beta-cell failure, type 2 diabetes mellitus, high-carbohydrate/high-fat, alloxan

## Abstract

To determine whether treatment with ginseng oligopeptides (GOPs) could modulate hyperglycemia related to type 2 diabetes mellitus (T2DM) in rats induced by high-fat diet and low doses of alloxan, type 2 diabetes was induced in male Sprague–Dawley (SD) rats by injecting them once with 105 mg/kg alloxan and feeding them high-carbohydrate/high-fat diet with or without GOP administration (0.125, 0.5, and 2.0 g/kg Body Weight) for 7, 24, and 52 weeks. Oral glucose test tolerance (OGTT), plasma glucose, serum insulin, level of antioxidant, and beta cell function were measured. Morphological observation and immunohistochemistry study of insulin of islets was performed by light microscopy. The insulin level and the expression of NF-κB and Bcl-2 family in pancreatic islets were also detected by Western blot analysis. In addition, survival time and survival rate were observed. After the treatment, the abnormal OGTT were partially reversed by GOPs treatment in diabetic rats. The efficacy of GOPs was manifested in the amelioration of pancreatic damage, as determined by microscopy analysis. Moreover, GOPs treatment increased the normal insulin content and decreased the expression of the NF-κB-signaling pathway. Compared with those in the control model, the survival time and rate were significantly longer. It is suggested that GOPs exhibit auxiliary therapeutic potential for diabetes.

## 1. Introduction

Diabetes is a worldwide health problem. A report from the World Health Organization suggests that diabetes affected up to 422 million adults worldwide in 2014, with type 2 diabetes mellitus (T2DM) the most common [1,2]. An important characteristic often associated with obesity and physical inactivity, insulin resistance precedes the development of hyperglycemia in subjects that eventually develop T2DM [3,4]. Nevertheless, the failure of pancreatic beta cells to secrete sufficient insulin in order to compensate for obesity and insulin resistance is the primary defect in T2DM [5,6]. Therefore, for the control of this process, effective therapeutic strategies should be developed that to prevent or delay the development of pancreatic beta cell dysfunction and death.

Insulin resistance, beta cell dysfunction, and apoptosis are factors in the pathogenesis of T2DM. Accumulating evidence based on studies in vivo and in vitro have demonstrated that oxidative stress-related production, chronic inflammation, and associated pro-inflammatory cytokines are risk factors that can trigger beta cell apoptosis in the pancreatic islets, leading to beta cell dysfunction in the pathogenesis of T2DM [7,8,9]. Modulation of peroxidation and pro-inflammatory parameters, as well as the inactivation of apoptosis-related components, such as Bcl-2, Bax, and caspase-3, have been shown to benefit patients with T2DM by using nutrition interventions [10,11,12]. Thus, nutritional therapy designed to reduce the sensitivity of beta cells to apoptosis triggers and block cytokine-signaling pathways may effectively protect the beta cells from apoptosis-related beta cell dysfunction, thereby benefiting patients with T2DM.

*Panax ginseng* Meyer (ginseng) is a traditional Chinese medicinal herb that has been widely used for thousands of years [13]. Owing to numerous regulatory chemicals, ginseng roots exhibit biological functions, antitumoral, antioxidant, and immunomodulatory effects as well as the normalization of the human metabolic system [14,15,16]. Several controlled clinical trials have demonstrated the therapeutic potential of ginseng for glycemic control [17]. In addition, a growing database of cell culture and animal studies indicates that ginseng may alleviate hyperglycemia by enhancing pancreatic beta cell function and reducing insulin resistance [18]. In 2012, the Ministry of Health of China approved ginseng and its products as food. Similarly, ginseng has been regarded as food for several years in many countries, including the United States, Canada, Japan, and Korea. Numerous functionality studies on the whole ginseng root have been conducted. Orally administered ginseng is not easily absorbable and digestible in the intestine. With the advances of technology, various bioactive substances extracted and discussed. Ginsenoside and polysaccharides are currently the most studied. Meanwhile, ginseng oligopeptides (GOPs) are a kind of nutrients with low molecular weight, high bioavailability, and absorption features, which have numerous potential physiological functions [19,20]. However, studies on the beneficial effects of ginseng oligopeptides (GOPs) on glycemia are rarely conducted.

In the present study, we used type 2 diabetic rats induced with low-dose alloxan and high-carbohydrate/high-fat diet to determine whether long-term GOPs administration would improve hyperglycemia.

## 2. Materials and Methods

### 2.1. Materials and Reagents

The GOP sample was derived from the roots of *Panax ginseng* Meyer planted in Jilin province, China. In addition, it was provided by Jilin Taigu Biological Engineering Co., Ltd. (Jilin, China). Identification of GOP were showed that 95.42% of the GOP sample had a molecular weight between 180 and 1000 Dalton. Amino acids accounted for 3.94% [20].

Assay kit used for the determination of alanine aminotransferase (ALT), aspartate aminotransferase (AST), serum urea nitrogen (SUN), total cholesterol (TC), triglyceride (TG), insulin and glycated serum protein (GSP), were purchased form Yingkexinchuang Science and Technology Ltd. (Macau, China). The detection kits of (Scr), superoxide dismutase (SOD) and malondialdehyde (MDA) were purchased from Nanjing Jiancheng Biotechnology Institute (Nanjing, China). Alloxan were from Sigma-Aldrich (St. Louis, MO, USA). The antibodies for Bax, Bcl-2, cleaved Caspase-3 and NF-κB were obtained from Santa Cruz Biotechnology (Santa Cruz, CA, USA). The insulin antibody was from Cell Signaling Technology (Danvers, MA, USA). The immunohistochemistry kit were from Beijing Zhongshan Golden Bridge Biotechnology Co. Ltd. (Beijing, China). All other reagents used in this study were of analytical grade.

Basal diet (AIN-93G diet) and high-carbohydrate/high-fat diet (66% basal diet, 15% lard, 10% plantation white sugar, 6% casein and 3% yolk powder) were produced by Beijing Keao Xieli Co. Ltd. (Beijing, China).

### 2.2. Animals and Gop Treatment

All animals were handled in accordance with the guidelines of the Principle of Laboratory Animal Care (NIH publication No. 85-23, revised 1985) of the Peking University Animal Research Committee (www.lab.pku.edu.cn, Ethical approval code: LA2015081, February 2015). A total of 150 male SD rats (5 weeks old, 130–170 g) were used in this treatment. Rats were obtained from the Animal Service of Health Science Center, Peking University, Beijing, and they were housed two per plastic cages with free access to chow and tap water in a SPF (Specific Pathogen Free) filter-protected air-conditioned room. The SPF condition was controlled temperature (21–25 °C), relative air humidity (50 ± 5%), and 12-h light/dark cycles (light on 07:30–19:30 h).

After acclimatization for one week, rats were food restricted and were given only water to drink for 6 h. Blood samples for plasma glucose were then collected from snipped tails by tail milking at 0, 30 and 120 min after administration of d-glucose (20% solution; 2 g/kg BW) by stomach tube. The level of plasma glucose was recorded as baseline. According to the baseline plasma glucose, the rats were randomly divided into five experimental groups (*n* = 30): normal control group (NC), model control group (MC) and three GOPs intervention groups which were designated as a low-dose group (GOP-L), medium-dose group (GOP-M), and high-dose group (GOP-H). GOP were accordingly administered to the mice of these three GOP intervention groups at 0.125, 0.500, and 2.00 g/kg BW, respectively, whereas the control were received 10 mL/kg distilled water). The doses setting were refer to the previous study in our lab [19,20].

### 2.3. Experimental Protocols

**Figure 1:**
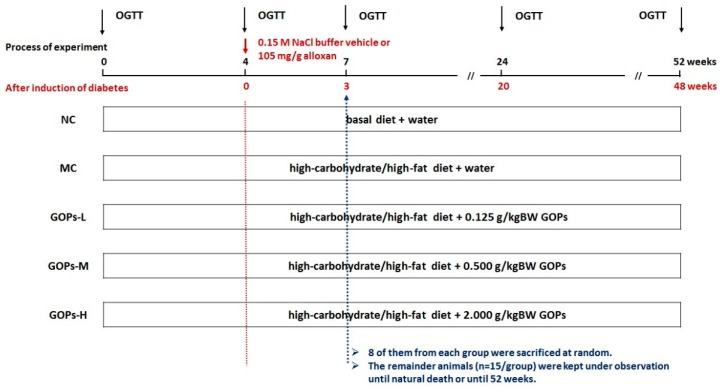
Schematic representation of the experimental procedures. The present study was performed in Sprague–Dawley rats made diabetic with low dose alloxan and a high-carbohydrate/high-fat diet. GOPs: *Panax ginseng* oligopeptide; OGTT: oral glucose tolerance test. NC, normal control group; MC, model control group; GOPs-L, low dose of GOPs-treated group; GOPs-M, medium dose of GOPs-treated group; GOPs-H, high dose of GOPs-treated group.

Rats were induced by the high-carbohydrate/high-fat diet as described previously for 4 weeks. In addition, then rats were food restricted and were given only water to drink for 24 h. Rats in the diabetes group were induced by 1 injection of 105 mg/kg BW alloxan dissolved in 0.15 M NaCl, whereas the rats in NC group were injected only 0.15 M NaCl. The blood glucose level was checked before and 72 h after alloxan injection to confirm the development of diabetes. Animals with blood glucose levels ≥300 mg/dL were selected for the study. Body weight and food consumption were recorded weekly. Experimental rats were administrated by gavage for 7 weeks, eight of them from each group were sacrificed at random. The remainder animals (*n* = 15/group) were kept under observation until natural death or until 52 weeks. All animals were observed three times daily, at 08:00, 14:00, and 20:00 hours, for morbidity and mortality. Observations included changes in skin, fur, eyes, mucous membranes, somatomotor activity, and behavior patterns. The method was referred to the previous study in our lab [21]. The survival time were recorded, and the survival rate were calculated (Figure 1).

They were anesthetized by CO_2_ inhalation and then sacrificed. Blood was obtained from femoral artery; serum was separated (3000 g for 20 min at 4 °C) for biochemical assays. Portions of the tissue for histopathological and western-blot examination were obtained. The tissue remaining were frozen in liquid nitrogen, and then saved in −80 °C. Pancreas were carefully excised and rinsed in ice-cold saline. After removing the excess water on the surface with filter paper, pancreas was weighed and pancreas/BW ratio was evaluated. Then part of pancreas was fixed in 4% paraformaldehyde for hematoxylin/eosin (HE) immunohistochemically staining. Additionally, a small portion of the same pancreas region from each group was immersed overnight in 2.5% glutaraldehyde (pH 7.4) in 0.1 mol/L phosphate buffered saline (PBS) at 4 °C. The manuscript was according to our previous study [12].

### 2.4. Biochemical Assay and Oral Glucose Test Tolerance (OGTT)

All detection kits were purchased from Beyotime Institute of Biotechnology (Beijing, China). Fasting serum insulin (FINS) and GSP levels were measured by using an enzyme-linked immunosorbent assay (ELISA) kit according to their manufacturer’s instruction.

The levels of ALT, AST, Scr, BUN, serum total proteins (TP), albumin (ALB), total cholesterol (TC), and triglyceride (TG) in serum were detected by Olympus AU400 automatic biochemistry analyzer (Olympus, Tokyo, Japan). The SOD activity, MDA and GSP levels in serum were determined with SOD, MDA and GSP detection kits according to the manufacturer’s protocols.

The OGTT procedure was performed at the end of 0, 3, 20 and 48 weeks after induction of diabetes. After food restricted for 6 h, blood samples were collected from snipped tails by tail milking at 0, 30, 60, and 120 min after administration of d-glucose (20% solution; 2 g/kg BW) by stomach tube. 1, 3, 20 and 44 weeks after induction of diabetes, fasting plasma glucose (FBG) and fasting serum insulin (FINS) were determined as described previously [12]. The OGTT-area under curve (OGTT-AUC) was calculated by the following formula:

OGTT-AUC = 0.25 × (0 h FBG (mmol/L) + 4 × 0.5 h FBG (mmol/L) + 3 × 2 h FBG (mmol/L)).

Homoeostasis model assessment (HOMA) of beta-cell function (HOMA-B) and assessment-insulin resistance (HOMA-IR) were calculated by the HOMA method using the following equations:

HOMA-B = (20 × FINS (μIU/mL))/(FBG (mmol/L) − 3.5).

HOMA-IR = FBG (mmol/L) × FINS(μIU/mL)/22.5

### 2.5. Western-Blot Assay

Western blots were carried out for NF-κB, Bax, cleaved Caspase-3 and Bcl-2. Nuclear and cytoplasmic proteins were extracted by ProteoJET™ Cytoplasmic and Nuclear Protein Extraction kit (Fermentas International Inc., Burlington, ON, Canada). Cells were scraped, pelleted by centrifugation at 250× *g* for 5 min, mixed with cell lysis buffer, homogenized, and set on ice for 10 min. Then, the mixture was centrifuged at 500× *g* for 7 min at 4 °C, and then the supernatant was further centrifuged at 20,000× *g* for 15 min at 4 °C. The supernatant of 20,000× *g* spin was collected as cytoplasmic protein extract, and the pellet of 500× *g* spin was washed and lysed with reagent of the kit and centrifuged at 20,000× *g* for 5 min at 4 °C; this final supernatant was collected as nuclei protein extract. Other protein samples were prepared as follows: harvested cells were homogenized in lysis buffer (10 mM Tris, pH 8.0, 120 mM NaCl, 0.5% NP-40, 1 mM EDTA, and protease inhibitors) for 30 min on ice and centrifuged at 10,000× *g* at 4 °C for 30 min [12]. The supernatants were then collected as samples. Protein content was examined by a BCA protein assay kit (Pierce Biotechnology, Rockford, IL, USA), according to the manufacturer’s instructions. Equal amounts of protein were separated by 10% SDS-PAGE and transferred to polyvinylidene fluoride membranes. After being blocked with 5% nonfat milk for 1 h, the membranes were incubated overnight at 4 °C with primary antibodies for cleaved Caspase-3 (1:100), NF-κB (1:1000), Bax (1:100), Bcl-2 (1:100) and β-actin (1:100). Goat anti-rabbit IgG (1:4000) as the secondary antibody was used. The blots were detected with Super ECL Plus Detection Reagent (Applygen, Beijing, China). In addition, Image Pro Plus 6.0 software (Media Cybernetics Inc., Bethesda, MD, USA) was used to perform densitometric analysis. Values were corrected with β-actin as a control.

### 2.6. Histopathology

For light microscopy, the fixed tissue samples were dehydrated through a graded ethanol series, embedded in paraffin and cut into 7 μm-thick sections with HE stain using a routine protocol. The stained sections were then observed from ×100 to ×400 magnifications.

### 2.7. Immunohistochemistry

Paraffin sections were deparaffinized, hydrated, and steamed in citrate buffer for 5 min for antigen retrieval. Endogenous peroxidase activity was inhibited using 3% hydrogen peroxide in methanol for 10 min. The sections were then incubated following the instructions in the commercial manual. Briefly, sections were blocked with protein-blocking agent followed by incubation with primary antibodies (1:200) at 4 °C overnight. Then they were incubated in turn with biotinylated secondary antibodies, with streptavidin peroxidase reagent, and with 3,3-diaminobenzidine for color development. Counterstaining was carried out with hematoxylin. Each step was separated by careful washings in PBS buffer. Slides were then analyzed from ×100 to ×400 magnifications by blinded pathologists under a light microscope.

### 2.8. Statistical Analysis

Statistical analyses were performed using SPSS software (version 19.0, SPSS Inc., Chicago, IL, USA). Variances of measurement data were checked for homogeneity by Bartlett’s test. When the data were homogeneous, the one-way analysis of variance test and LSD methods were used. Tamhane’s T2 test is used after data are transformed to analyze data among multiple groups if variances are unequal. Survival curves of rats were estimated with Kaplan-Meier analysis, and differences between groups were compared with two-sided log-rank test. All reported *p* values were two-sided. A value of *p* < 0.05 was considered significant.

## 3. Results

### 3.1. Effect of Long-Term GOPs Administration Could Bring a Survival

**Figure 2:**
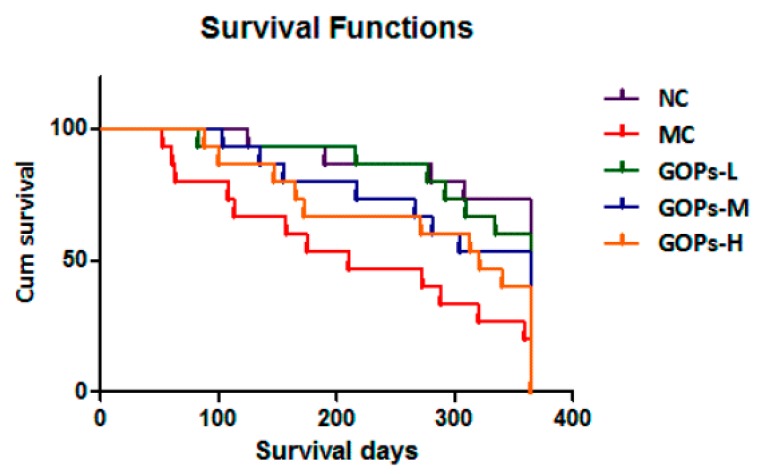
Effects of *Panax ginseng* oligopeptide (GOPs) on survival time in rats. 15 rats/group were used in each group (log-rank test, χ^2^ = 12.706, *p* = 0.013). NC, normal control group; MC, model control group; GOPs-L, low dose of GOPs-treated group; GOPs-M, medium dose of GOPs-treated group; GOPs-H, high dose of GOPs-treated group.

Generally (shown in Figure 2), the rats in NC group were in good physical and mental state. On the contrast, the diabetic rats were skinny with gloomy and frizzier in depression and laziness. After 52 weeks, GOPs-treated rats were in a better condition compared with the rats of MC group. The first death of rats was on Day 147 (in NC group) and Day 52 (in MC group). The number remaining alive at the end of the study in the 5 groups was 11 (with 73.3% survival rate), 3 (with 20% survival rate), 9(with 60% survival rate), 8 (with 53.3% survival rate) and 6 (with 40% survival rate) respectively of NC, MC and 3 GOPs intervention groups. As shown in Figure 2, GOPs prolonged the mean life span days of rats (log-rank test, χ^2^ = 12.706, *p* = 0.013). It should be noted that diabetic rats had a much shorter life span (327.13 ± 18.52 days for NC and 217.93 ± 30.36 days for MC; log-rank test, χ^2^ = 9.253, *p* = 0.002). The significant effect of GOPs on longevity was attenuated, because of the smaller sample size (GOPs-L vs. MC, χ^2^ = 6.435, *p* = 0.011; GOPs-M vs. MC, χ^2^ = 3.581, *p* = 0.058 and GOPs-H vs. MC, χ^2^ = 1.685, *p* = 0.194).

### 3.2. Effect of GOPs on Body Weight

As shown in Figure S1, the trend of body weight in MC group was increased first increased (for the first 20 weeks), and then started to drop. Comparing with the rats of NC group, the body weight of rats in MC group was significantly lower (*p* < 0.05), whereas it was higher in three GOPs-treated groups (*p* < 0.05, after 28 weeks, among the GOPs groups). At week 7, there were significantly differences among the groups (*p* = 0.00 for NC and MC; *p* < 0.047 for GOPs and MC; *p* = 0.03 for GOP-H and NC).

### 3.3. Effect of GOPs on OGTT and Insulin Metabolism

**Figure 3:**
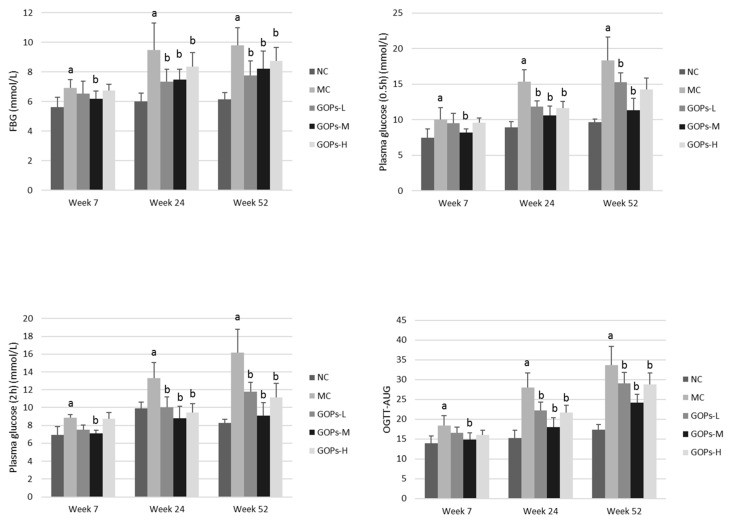
Effect of GOPs on OGTT in normal and diabetic rats. 8 rats/group (at week 7) and 6 rats/group (at week 52) were used in each group. The data were analyzed for significance of differences by one-way analysis of variance test. ^a^ *p* < 0.05 versus NC rats, ^b^ *p* < 0.05 versus MC rats. FBG, fasting blood-glucose; GOPs, *Panax ginseng* oligopeptides; OGTT, oral glucose tolerance test; OGTT-AUC, OGTT-area under curve. NC, normal control group; MC, model control group; GOPs-L, low dose of GOPs-treated group; GOPs-M, medium dose of GOPs-treated group; GOPs-H, high dose of GOPs-treated group.

The result of OGTT and OGTT-AUC are illustrated in Figure 3, as well as the results of GSP, FINS, HOMA-IR and HOMA-B are shown in Table 1. As the growth of age, the difference among the groups became more and more significant. At week 52, the FBG levels were 7.76 ± 0.98 (GOP-L), 8.23 ± 1.16 (GOP-M) and 8.73 ± 0.9 (GOP-H), showed a lower level compared to the MC group (9.80 ± 1.19). The values of OGTT-AUC in the GOPs groups were 29.06 ± 2.74, 24.12 ± 2.15 and 28.75 ± 2.92, respectively, which were significantly lower than that of MC groups (33.68 ± 4.72). At week 52, the GSP levels reduced by 16.13% (*p* < 0.05), 11.91%, and 13.65% (*p* < 0.05) in GOPs relative to that of MC group, respectively. The decrease in the GSP level of GOP-L group was more pronounced than that in MC group, thereby indicating that the treatment with GOPs effectively reduces blood and GSP in T2DM rats. The FINS level of the MC group was significantly higher than that in NC group (*p* < 0.05 for both week 7). Relative to the MC group, the FINS of GOPs groups were significantly reduced (*p* < 0.05 for both week 7). However, there were no significantly differences among the groups at week 52 (*p* > 0.05). The HOMA-IR value of MC group was significantly higher than that in NC group, whereas the HOMA-B level was remarkable lower (*p* < 0.05 for both week 7 and 52). After GOPs treatment, the HOMA-IR values were markedly lower, conversely HOMA-B was higher (*p* < 0.05 for both week 7 and 52). These finding indicated that GOPs could effectively decrease the FINS and HOMA-IR, whereas increase the HOMA-B values in T2DM rats.

### 3.4. Effect of GOPs on NF-κB Activation in the Pancreas of Diabetic Rats

**Figure 4:**
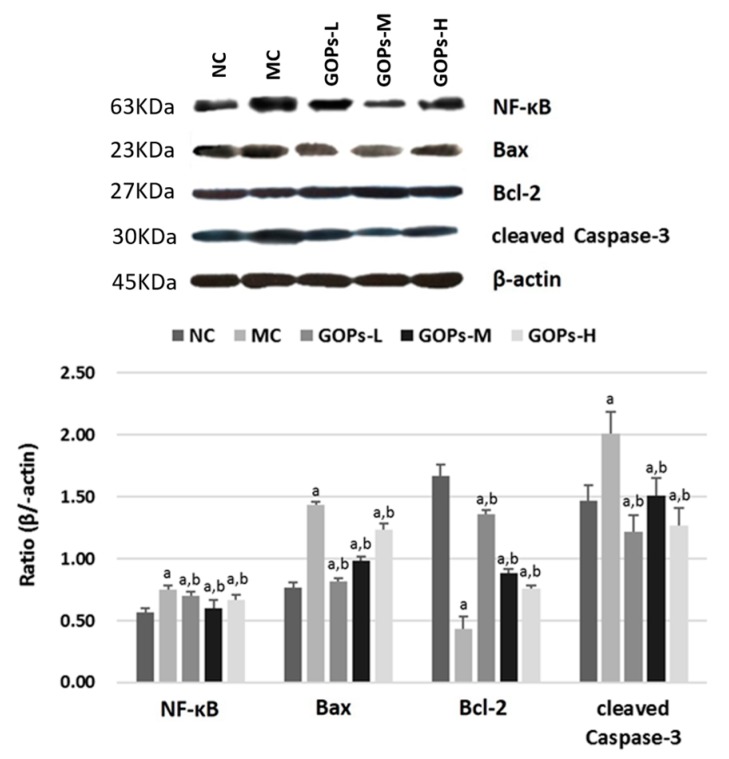
Effect of *Panax ginseng* oligopeptide (GOPs) on NF-κB, Bax, Bcl-2 and cleaved Caspase-3 activation in the pancreas of normal and diabetic rats at week 7. 8 rats/group were used in each group. beta-actin protein levels were used as a control. The ratios were calculated as means ± SEM of three determinations from three individual rats in each group. ^a^ *p* < 0.05 versus NC rats, ^b^ *p* < 0.05 versus MC rats. NC, normal control group; MC, model control group; GOPs-L, low dose of GOPs-treated group; GOPs-M, medium dose of GOPs-treated group; GOPs-H, high dose of GOPs-treated group.

The alternations of NF-κB, Bcl-2, Bax and cleaved Caspase-3 in pancreas in various treatment groups are shown in Figure 4. A significant decrease in NF-κB (*p* < 0.05), Bax (*p* < 0.05) and cleaved Caspase-3 (*p* < 0.05) expression of MC, whereas increased in Bcl-2 (*p* < 0.05) expression of MC and GOPs groups were observed compared to that in controls at 7 weeks.

### 3.5. Effect of GOPs on Panceatic Histopathology and Insulin Expression in Pancreatic Islets

**Figure 5:**
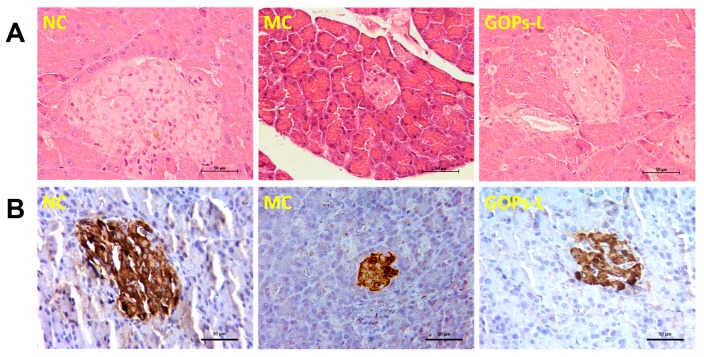
Effect of GOPs on pancreatic histopathology (**A**) and immunohistochemistry study (**B**) on insulin of islets in normal and diabetic rats. 6 rats/group were used in each group. NC, normal control group; MC, model control group; GOPs-L, low dose of GOPs-treated group; Scale bar = 50 μm.

Histopathological observations were shown in Figure 5A. Pancreatic histology of normal control rats was normal throughout the whole study. On the contrary, pancreas showed evidence of severe damage characterized by reduced pancreatic islet area in diabetic control rats. The atrophied pancreatic islets were ameliorated in GOPs treated diabetic rats.

Figure 5B demonstrated the level of insulin in pancreatic islets of each group. Healthy pancreatic islets in normal groups exhibited diffused staining with brown or yellow granules. In marked contrast, the insulin expression in diabetic pancreas decreased characterized by the depletion of brown or yellow granules. Administration of GOPs to diabetic rats increased the level of insulin when compared with diabetic control rats.

### 3.6. Effect of GOPs on SOD Activity and MDA Level

As shown in Figure S2, at week 7, low levels of serum MDA were detected in NC rats. However, the levels of MDA in the MC group were near twice-fold higher than that in NC group, reflecting the oxidative stress-related high levels of serum MDA were significantly reduced in GOPs-treated lipid peroxidation. Serum SOD revealed that the level of SOD in MC group were significantly lower than that in NC group. However, serum SOD, in the GOPs-treated rats, were significantly elevated in higher level, near to that of the NC rats, as compared with that in the NC group.

### 3.7. Effect of GOPs on Liver and Kidney Function

**Figure 6:**
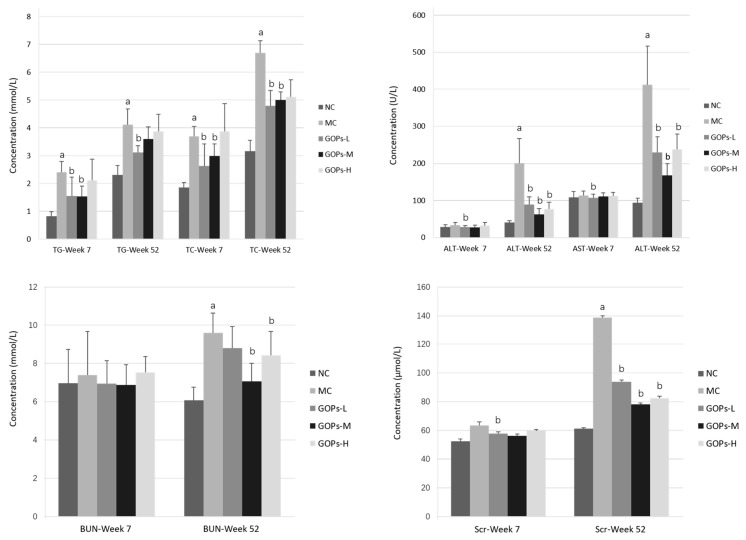
Effect of GOPs on liver and kidney profiles in normal and diabetic rats. 8 rats/group (at week 7) and 6 rats/group (at week 52) were used in each group. The data were analyzed for significance of differences by one-way analysis of variance test. ^a^ *p* < 0.05 versus NC rats, ^b^ *p* < 0.05 versus MC rats. ALB, albumin; ALT, alanine aminotransferase; AST, aspartate aminotransferase; BUN, blood urea nitrogen; GOPs, *Panax ginseng* oligopeptides; Scr, serum creatinine; TC, total cholesterol; TG, triglyceride. NC, normal control group; MC, model control group; GOPs-L, low dose of GOPs-treated group; GOPs-M, medium dose of GOPs-treated group; GOPs-H, high dose of GOPs-treated group.

The lipid, liver and kidney profiles of each group are shown in Figure 6. Both at the 7 and 52 weeks of the study, the TC (*p* < 0.05 for 7 and 52 weeks), TG (*p* < 0.05 for 7 and 52 week), ALT (*p* < 0.05 for 52 weeks), AST (*p* < 0.05 for 52 weeks), BUN (*p* < 0.05 for 52 weeks) and Scr (*p* < 0.05 for 52 weeks) levels of the diabetic rats significantly increased compared with the rats in NC group, whereas these profiles were decreased in the GOPs groups in the comparison of MC group. However, there was no remarkable differences in ALB and GLB levels (*p* > 0.05 for 7 and 52 weeks), between the normal and diabetic rats.

## 4. Discussion

Diabetes, one of the three killer diseases, exhibits a sharply increasing prevalence. In the clinical setting, ginseng and its extract components used as traditional Chinese medicine exert inhibitory effects on all severe side effects after prolonged treatment. In the present study, the effects of GOPs on hyperglycemia, insulin metabolism, oxidative stress, and expression of beta-cell apoptosis-related protein were evaluated for the first time.

Studies have suggested that extreme nutritional factors usually induce insulin resistance (IR) [22,23]. Alloxan, a classical diabetogen, can be selectively toxic to the pancreatic beta cells through oxidative stress-related DNA damage in the sensitive cells. Alloxan-induced rats have been widely used for screening and estimating animal models for the evaluation of anti-diabetic agents [24]. Whereas treatment with low doses of alloxan commonly causes inflammation and partial loss of beta cells as well as beta cell-specific autoimmunity in animals, treatment with high doses of alloxan usually induces extensive beta cell death and insulin deficiency [25]. Compared with the control rats, the diabetic rats exhibited significantly higher fasting blood glucose levels, poorer oral glucose tolerance test results, higher glycosylated serum protein (GSP) levels, more serious insulin resistance, and more severe pancreatic impairment.

GOP treatment significantly reduced GSP and corrected hyperglycemia in diabetic rats. The GSP levels can effectively reflect the blood glucose levels over a period of time [26]. Insulin resistance characterizes T2DM; thus, decreasing insulin resistance is a vital approach to treating T2DM. GOP treatment significantly modulated the levels of serum insulin at weeks 7 and 52 as significant difference was indicated the FINS, HOMA-IR, and HOMA-B levels, compared with the MC groups. Diabetes is a progressive disease, and glucose levels are known to increase with age. Notably, with age, the FINS and HOMA-B levels decreased, whereas the FBG and HOMA-IR levels increased in diabetic rats; these effects are consistent with previous age-related studies [27]. Moreover, there are findings of this study that GOPs ameliorated alloxan-induced islet deterioration and insulin deficiency in rats by histopathological and immunohistochemistry study.

The mechanism underlying these anti-diabetic effects requires investigation. Apoptosis was caused by physiologic and pathologic signals. Through the regulation of death-related genes, death-receptor pathways were activated, including Bcl-2, Bax, and the caspase pathway [28,29]. To elucidate the mechanism of action, we analyzed Bax, Bcl-2, and cleaved Caspase-3 expression by Western blot analysis at week 7. Bcl-2 and Bax, which are typical proteins of the Bcl-2 family, play key roles in caspase-dependent apoptosis. Bcl-2 is an anti-apoptotic protein mainly located in the nuclear membrane and the mitochondrial membrane; the family member Bax, which promotes apoptosis, is mainly located in the cytoplasm. They change the mitochondrial membrane permeability and trigger the activation of caspases that lead to apoptosis [30]. GOPs prevented beta cells from undergoing apoptosis by increasing the expression of anti-apoptotic Bcl-2 and decreasing the expression of pro-apoptotic Bax and caspase-3. Therefore, increasing Bcl-2 expression and decreasing caspase-3 expression can be the potential mechanism for the anti-apoptotic effect of GOPs. The current study is the first to report that GOPs exert an anti-apoptotic effect by regulating Bcl-2, Bax, and caspase-3 expression in vitro. To further investigate the mechanism of action, we determined the NF-κB protein expression. NF-κB is an important transcription factor that binds to DNA regulatory sequences in cells and controls the rate of gene expression. Our previous experiments [19] indicated that GOPs downregulated the synthesis and secretion of cytokines and chemokines, such as Il-6, Il8, and Tnf, via NF-κB, similar to the anti-inflammatory mechanism of GOPs. Moreover, inflammatory cytokines may trigger beta-cell death via an NF-κB-dependent path, resulting in caspase activation and eventually, cell failure [8]. These results agree with the conclusion that GOPs inhibits NF-κB activity to protect against inflammatory of diabetes. To our knowledge, this experiment is the first to show that GOPs inhibits inflammatory damage in diabetes via the inactivation of the NF-κB and Bcl-2 family-signaling pathways.

Oxidative stress, as critical factor in pathogenesis of T2DM, can change in the mitochondrial membrane potential and the release of cytochrome c by promoting the production of reactive oxygen species (ROS) and reactive nitrogen species (RNS), thus triggering cell apoptosis. A high-fat diet can induce hyperlipidemia and associated oxidative stress [31]. We evaluated the effect of GOP treatment on systemic levels of oxidative stress and found significantly increased MDA levels, indicating lipid peroxidation, and decreased SOD levels in the MC group of rats. However, following the GOP treatment, the levels of serum MDA significantly decreased, whereas the levels of antioxidant SOD markedly increased in the GOP-treated rats than in the MC group of rats. Results indicated that GOP treatment inhibited hyperglycemia-related oxidative stress in diabetic rats. Animal studies showed that high levels of ROS and RNS were risk factors for beta cell apoptosis [32,33]. In addition, oxidative stress in beta cells reduces the mitochondrial transmembrane potential, promoting the release of cytochrome c into cytosol and activating caspase-3; the Bcl-2 family regulates this apoptotic process [34]. Therefore, the reduced serum MDA levels and elevated SOD levels attributed to GOP treatment may also protect pancreatic beta cells from T2DM-related apoptosis.

An interesting finding in this study relates to the survival time of diabetic rats. Diabetes is associated with severe secondary complications including heart attack, kidney failure, leg amputation, vision loss, stroke and nerve damage largely caused by poor glycemic control. They can also increase the overall risk of premature death. The results we obtained indicate that GOPs can significantly enhance and prolong the survival of diabetic rats. The benefit was partially attributed to amelioration of impaired insulin secretion and liver and kidney function in diabetes.

This study includes certain limitations. (1) There is a low survival rate in NC group (73%), owing to the rats in NC group were intragastrically administered 10 mL/kg distilled water every day, which may bring some damage to the animals; (2) The small sample size prevented the analysis of the expression of apoptosis-related protein at year 1. We did not investigate the rats throughout their lifetime. Because of that, we couldn’t draw the effect of GOPs on life span in diabetic rats; (3) Moreover, we did not do immunohistochemistry in this study, such as terminal deoxynucleotidyl transferase-mediated dUTP biotin nick end labeling (TUNEL) staining. The factors to which the prolonged survival days may be attributed were not identified. These concerns can be topics for further study.

## 5. Conclusions

This study demonstrate that GOPs treatment significantly reduced FBG in diabetic rats. GOPs treatment significantly inhibited inflammation via the downregulation of NF-κB expression. Similarly, oxidative stress was improved in the GOPs treatment group. It is indicated the effect of GOPs treatment on T2DM can be potentially mediated by inhibiting oxidative stress and Bcl-2 family expression. Our findings indicated that the beneficial effect of GOPs in glucose metabolism, which may aid in the design of a new therapy for T2DM in humans.

## Figures and Tables

**Table 1:** Effect of GOPs on serum FINS, GSP, HOMA-IR and HOMA-B levels in normal and diabetic rats.

Parameter		NC	MC	GOPs-L	GOPs-M	GOPs-H
FINS	Week 7	14.92 ± 2.6	28.48 ± 5.27 ^a^	16.47 ± 4.18 ^b^	14.99 ± 3.77 ^b^	22.53 ± 7.48
FINS	Week 52	12.34 ± 0.71	14.98 ± 0.98	13.67 ± 0.54	13.25 ± 0.69	13.21 ± 0.6
GSP	Week 7	1.95 ± 0.09	2.68 ± 0.12 ^a^	2.33 ± 0.24 ^b^	2.45 ± 0.09 ^b^	2.64 ± 0.15
GSP	Week 52	2.01 ± 0.08	4.03 ± 0.14 ^a^	3.38 ± 0.5 ^b^	3.55 ± 0.43 ^b^	3.47 ± 0.23 ^b^
HOMA-IR	Week 7	1.25 ± 0.21	2.80 ± 0.55 ^a^	1.42 ± 0.79 ^b^	1.61 ± 0.53 ^b^	2.05 ± 0.82
HOMA-IR	Week 52	3.31 ± 0.26	6.51 ± 0.45 ^a^	4.84 ± 0.44 ^b^	4.84 ± 0.44 ^b^	5.29 ± 0.28 ^b^
HOMA-B	Week 7	98.03 ± 12.75	46.53 ± 4.83 ^a^	62.82 ± 12.16 ^b^	57.6 ± 10.87	48.85 ± 9.85
HOMA-B	Week 52	69.79 ± 46.52	43.46 ± 15.51 ^a^	51.08 ± 8.7 ^b^	52.00 ± 10.82 ^b^	45.49 ± 11.18

8 rats/group (at week 7) and 6 rats/group (at week 52) were used in each group. The data were analyzed for significance of differences by one-way analysis of variance test. ^a^ *p* < 0.05 versus NC rats, ^b^ *p* < 0.05 versus MC rats. FINS, fasting serum insulin; GSP, glycated serum protein; GOPs, *Panax ginseng* oligopeptides; HOMA-IR, homeostasis model homeostasis model of assessment for insulin resistance index; HOMA-B, homeostasis model homeostasis model of assessment for beta-cell index. NC, normal control group; MC, model control group; GOPs-L, low dose of GOPs-treated group; GOPs-M, medium dose of GOPs-treated group; GOPs-H, high dose of GOPs-treated group.

## Supplementary Materials

The following are available online at www.mdpi.com/2072-6643/9/10/1061/s1, Figure S1: Growth curve for Sprague–Dawley rats treated with GOPs for 52 weeks; Figure S2: The serum activities of SOD and MDA level in Sprague–Dawley rats treated with GOPs for week 7.
