# Supplementary material for: Beneficial Effects of Small Molecule Oligopeptides Isolated from Panax ginseng Meyer on Pancreatic Beta-Cell Dysfunction and Death in Diabetic Rats
Source: Nutrients. 2017 Sep 26;9(10):1061. doi: 10.3390/nu9101061 (PMC5691678; doi:10.3390/nu9101061)
Supplement: Supplementary file 1 [file nutrients-09-01061-s001.zip › nutrients-202449-supplementary.pdf]

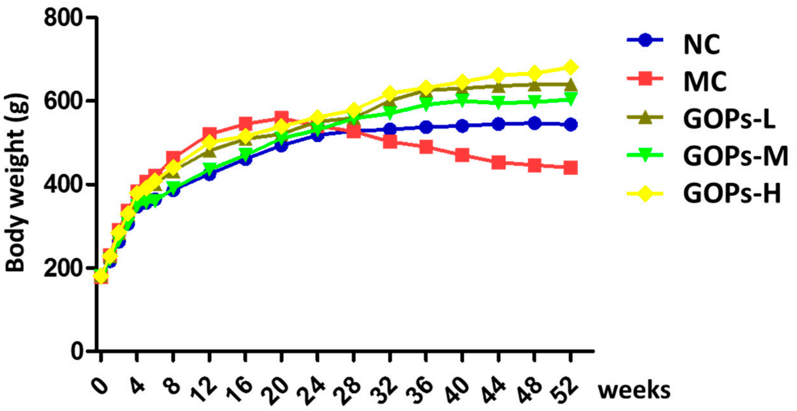

Figure S1. Growth curve for Sprague-Dawley rats treated with GOPs for 52 weeks. 15 rats/group were used in each group. The data were analyzed for significance of differences by one-way analysis of variance test. [a] $p < 0.05$ versus NC rats, [b] $p < 0.05$ versus MC rats. GOPs: *Panax ginseng* oligopeptide. NC, normal control group; MC, model control group; GOPs-L, low dose of GOPs-treated group; GOPs-M, medium dose of GOPs-treated group; GOPs-H, high dose of GOPs-treated group.

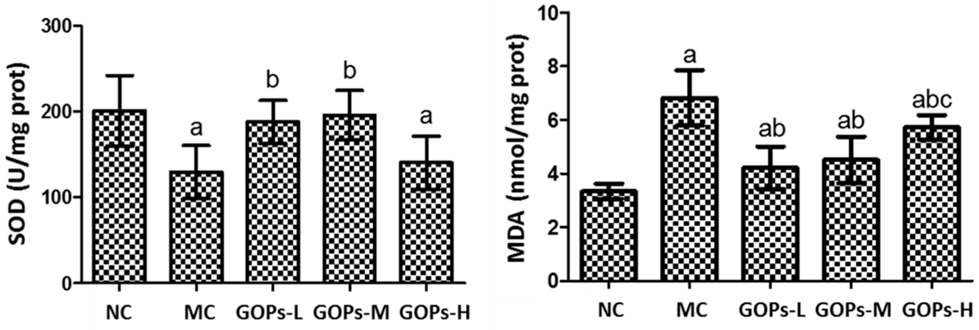

Figure S2. The serum activities of SOD and MDA level in Sprague-Dawley rats treated with GOPs for week 7. 8 rats/group (at week 7) were used in each group. The data were analyzed for significance of differences by one-way analysis of variance test. [a] $p < 0.05$ versus NC rats, [b] $p < 0.05$ versus MC rats. GOPs: *Panax ginseng* oligopeptide; MDA: malondialdehyde; SOD: superoxide dismutase. NC, normal control group; MC, model control group; GOPs-L, low dose of GOPs-treated group; GOPs-M, medium dose of GOPs-treated group; GOPs-H, high dose of GOPs-treated group.
